# Manipulation of Neutrophils by *Porphyromonas gingivalis* in the Development of Periodontitis

**DOI:** 10.3389/fcimb.2017.00197

**Published:** 2017-05-23

**Authors:** Maja Sochalska, Jan Potempa

**Affiliations:** ^1^Department of Microbiology, Faculty of Biochemistry, Biophysics and Biotechnology, Jagiellonian UniversityKrakow, Poland; ^2^Department of Oral Immunology and Infectious Diseases, School of Dentistry, University of LouisvilleLouisville, KY, United States

**Keywords:** periodontitis, neutrophils, *Porphyromonas gingivalis*, virulence factors, inflammation

## Abstract

The pathogenesis of the chronic periodontal disease is associated with a skewed host inflammatory response to periodontal pathogens, such as *Porphyromonas gingivalis*, that accounts for the majority of periodontal tissue damage. Neutrophils are the most abundant leukocytes in periodontal pockets and depending on the stage of the disease, also plentiful PMNs are present in the inflamed gingival tissue and the gingival crevice. They are the most efficient phagocytes and eliminate pathogens by a variety of means, which are either oxygen-dependent or -independent. However, these secretory lethal weapons do not strictly discriminate between pathogens and host tissue. Current studies describe conflicting findings about neutrophil involvement in periodontal disease. On one hand literature indicate that hyper-reactive neutrophils are the main immune cell type responsible for this observed tissue damage and disease progression. Deregulation of neutrophil survival and functions, such as chemotaxis, migration, secretion of antimicrobial peptides or enzymes, and production of reactive oxygen species, contribute to observed tissue injury and the clinical signs of periodontal disease. On the other hand neutrophils deficiencies in patients and mice also result in periodontal phenotype. Therefore, *P. gingivalis* represents a periodontal pathogen that manipulates the immune responses of PMNs, employing several virulence factors, such as gingipains, serine proteases, lipid phosphatases, or fimbriae. This review will sum up studies devoted to understanding different strategies utilized by *P. gingivalis* to manipulate PMNs survival and functions in order to inhibit killing by a granular content, prolong inflammation, and gain access to nutrient resources.

## Periodontitis

Periodontitis is an inflammatory disease, which affects tissues surrounding and supporting the teeth. It occurs in response to dysbiotic periodontal microbiota accumulating as a bacterial plaque on the tooth surface below the gum line that triggers a chronic inflammatory reaction (Graves et al., 2008). Dysbiotic bacteria, also called pahtobionts, are commensal microorganisms that under conditions of disrupted homeostasis have a potential to deregulate inflammatory responses and cause disease (Hajishengallis and Lamont, 2012). Chronic periodontitis is one of the most prevalent inflammatory diseases of humans and in its most severe form it is the sixth most prevalent disease affecting 11.2% of the global population and representing a substantial public health burden (Kassebaum et al., 2014). Risk factors for developing periodontal disease (PD) are genetic factors, cigarette smoking, diabetes, osteoporosis, age, and infections with red complex bacteria. Red complex bacteria is a group of three species including *Porphyromonas gingivalis, Treponema denticola*, and *Tannerella forsythia*, which presence is strongly associated with each other and disease sites (Socransky et al., 1998; Van Dyke and Sheilesh, 2005).

According to the well-accepted polymicrobial synergy and dysbiosis model, the host immune response is initially manipulated by keystone pathogens (e.g., *P. gingivalis*) with the help of accessory pathogens and is subsequently over-activated by pathobionts (Hajishengallis et al., 2015a). This concept of keystone species is derived from basic ecological studies. A keystone pathogen is a microorganism that exerts a huge impact on the microbiotic community, while being present at very small quantity (Darveau et al., 2012). Another hypothesis on the development of the oral diseases is based on the imbalance in the oral microflora caused by ecological stress, causing an enrichment of some periodontal pathogens (Marsh, 1994, 2003). This “Ecological Plaque Hypothesis” is supported by the fact that expressed by *P. gingivalis* gingipains degrade collagen (Houle et al., 2003) and therefore enrich the growth environment in peptides favoring growth of the red complex bacteria. A futile attempt of the host to eradicate periodontopathogens fuels a chronic inflammatory reaction in the infected periodontium. In genetically susceptible hosts, this leads to a dissolution of periodontal ligament, alveolar bone resorption, deep periodontal pocket formation, and eventual tooth loss (Reynolds and Meikle, 1997). The presence of keystone pathogens can cause deregulated inflammation and disease without apparent predispositions (Hajishengallis, 2014).

## Neutrophils' functions and survival

Neutrophils, also called polymorphonuclear leukocytes or, in short, PMNs, are the most abundant white blood cells in the gingival crevice and periodontal pocket, where they play a crucial role in the innate immunity response against bacterial infection and thus are responsible for the maintenance of homeostasis in periodontal tissues. PMNs are produced in the bone marrow in large amounts, meaning 5^−10^ × 10^10^ cells per day, and are released into the peripheral blood as terminally differentiated and fully competent effector cells (Borregaard, 2010). This is in contrast to adaptive immunity, where T and B lymphocytes require activation and proliferation steps in secondary lymphatic organs in order to become effector cells (Segal, 2005; Nathan, 2006).

**Figure 1:**
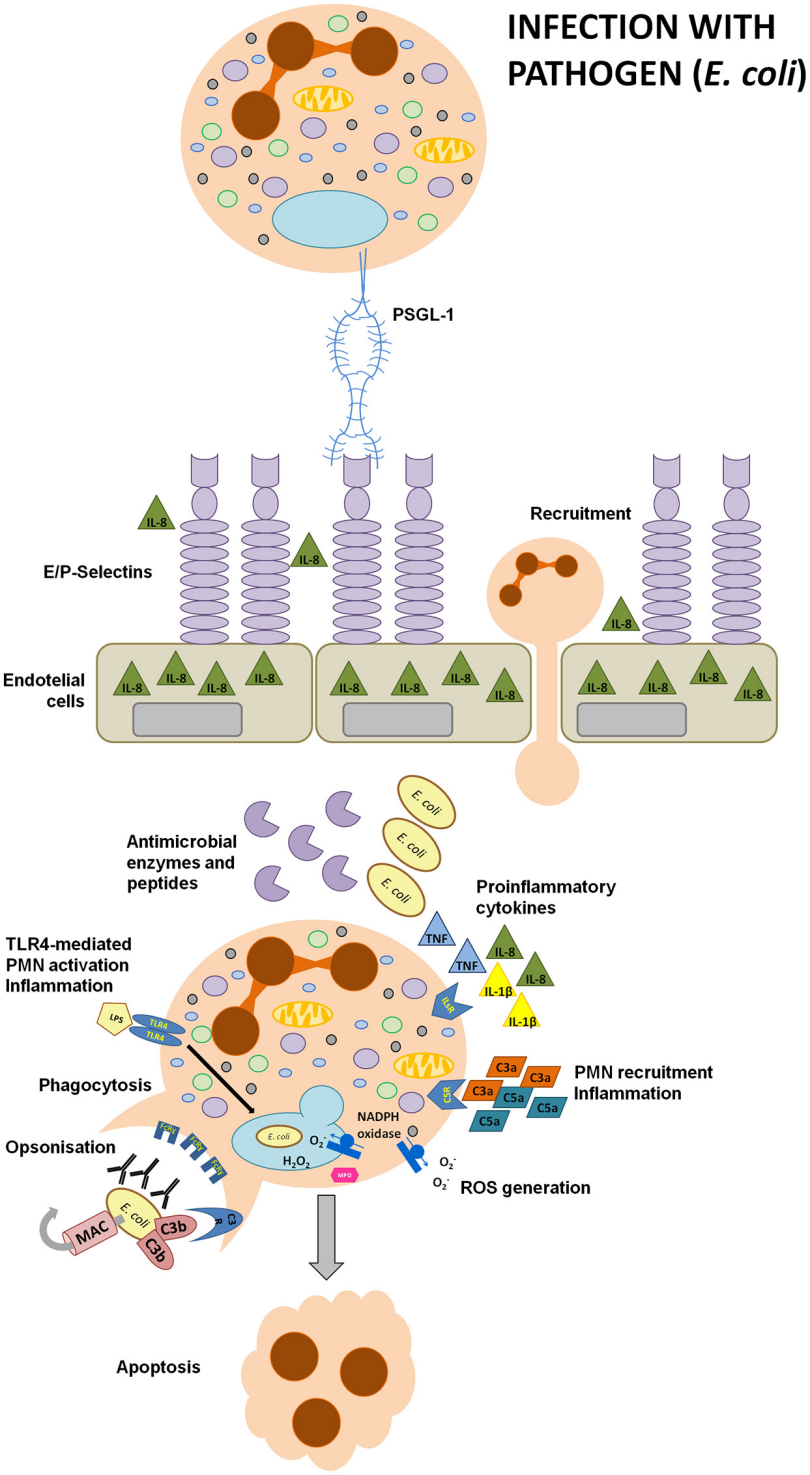
**Immune responses to pathogens**. During an infection with pathogens, for example *E. coli*, lipopolisaccharide enhances the secretion of chemotactic IL-8 and stimulates the upregulation of E- or P-selectins expression on gingival endothelial cells (GECs). Selectins facilitate neutrophils adhesion during transmigration as they interact with PSGL-1 expressed on PMNs. Moreover, the presence of microbes and their particles activates the complement cascade. C3a and C5a are anaphylatoxins with a strong chemotactic and pro-inflammatory potential. IgG and IgM antibodies or C3b recognize bacterial antigens and opsonize invading pathogens thus facilitating bacterial phagocytosis. LPS activates the TLR4 signaling pathway in recruited neutrophils, eliciting strong inflammatory responses designed to inactivate the pathogen. Inflammatory responses include the production of reactive oxygen species, secretion of pro-inflammatory cytokines and antimicrobial enzymes or peptides, such as cathepsin G, elastase, cathelicidins or defensins. After a successful bacterial clearance, neutrophils undergo apoptosis, an essential process triggering the resolution of inflammation.

Neutrophils are the most efficient phagocytes and they eliminate pathogens by a variety of means, which are either oxygen-dependent (oxidative burst) or oxygen-independent (anti-microbial peptides and lytic or proteolytic enzymes; Figure 1). Neutrophil priming by pro-inflammatory signals recruits the cytosolic NADPH oxidase complex to the phagosome membrane which leads to the generation of reactive oxygen species (ROS). The respiratory burst can disrupt bacterial phospholipid bilayers, degrade or inactivate proteins, and trigger DNA damage (Segal, 2005; Nauseef, 2007). Importantly, these processes can occur in hypoxic periodontal pockets, where oxygen concentration is as low as 1–3% (Loesche et al., 1988). In order to meet high-energy requirements, neutrophils engage glycolysis, which is a huge advantage under hypoxic conditions present in periodontal pockets. This unique strategy is in contrast to ATP production mechanisms in most cells in the human body (Borregaard and Herlin, 1982). Non-oxidative microbial killing relies on the contents of three types of cytoplasmic granules, namely: azurophilic (primary) granules, specific (secondary) granules, and gelatinase granules. Neutrophil activation triggers granule fusion with phagosomes. These granules deliver antimicrobial proteins and peptides, such as azurocidin, cathelicidin, α-defensins, lysozyme, lactoferrin, elastase, and cathepsin G, that disrupt bacterial cell envelope, destroy peptydoglican, degrade proteolytic bacterial virulence factors, or sequester iron (Soehnlein, 2009). Beside this antimicrobial arsenal, PMNs can additionally form neutrophil extracellular traps (NETs), which are composed of decondensed nuclear or mitochondrial DNA associated with antibacterial (granule) enzymes, peptides, and histones. These extracellular structures are designed to disable invading pathogens and elicit proinflammatory responses (White P. C. et al., 2016). PMNs have the shortest lifespan of all immune cells, i.e., around 24 h under the steady state, while for example T lymphocytes may stay alive for weeks. Normally, neutrophils circulate in the blood for 6–12 h and then home to the bone marrow, spleen or liver where they undergo apoptosis. Subsequently, they are phagocytosed by Kupffer cells in the liver or by red pulp macrophages in the spleen (Summers et al., 2010; Vier et al., 2016). This short life-span of neutrophils is tightly controlled by apoptosis, which is a form of programmed cell death relying on enzymes of the Caspase family of endopeptidases. It is a critical process involved in embryonic development or the maintenance of tissue homeostasis in the adult organism. Its deregulation is implicated in different pathologies, including cancerogenesis or disorders of the immune system (Sochalska et al., 2016; Tuzlak et al., 2016). Apoptosis is a very precise process controlled by the Bcl-2 family proteins, which encompasses many pro- and anti-apoptotic proteins that form homo- or heterodimers in order to promote or prevent apoptosis (Sochalska et al., 2015). The pro-survival family members, i.e., Bcl-2, Bcl-xL, Bcl-w, Mcl-1, and A1, share four BH (Bcl-2 homology) domains and beside A1, they possess a transmembrane domain at the C-terminal end. They prevent apoptosis by sequestering (inhibiting) pro-apoptotic BH3-only proteins, such as Bim, Bmf, Noxa, Puma, Bid, Bad, Bmf, and HRK. The BH3-only proteins act as sentinels for various stress stimuli, such as DNA damage, growth factor deprivation, ER-stress or oncogenic transformation (Tuzlak et al., 2016). Moreover, after successful phagocytosis of invading bacteria, neutrophils undergo apoptosis, a very important step for the resolution of inflammation, which is called phagocytosis-induced cell death (PICD). Exposure of the cell to an apoptotic stimulus frequently engages BH3-only proteins, either transcriptionally or translationally, which allows them to either directly (Bim and tBid) or indirectly (all BH3-only) activate the pro-apoptotic effector proteins Bax/Bak (Czabotar et al., 2014; Garcia Saez and Villunger, 2016).

However, in the context of *P. gingivalis* infection, neutrophils are unable to phagocyte this periodontopathogen present within the huge biofilm structure. During phagocytosis of something too big to be ingested, a process so-called “frustrated phagocytosis,” they generate reactive oxygen species and secrete enzymes in order to destroy the pathogen. Unfortunately, these secreted weapons concomitantly contribute to the inflammatory destruction of gingival tissues and alveolar bone (Scott and Krauss, 2012).

## Neutrophils in periodontitis

On one hand, functional neutrophils are indispensable for the maintenance of periodontal health, as illustrated by several neutrophil genetic defects, including neutropenias (Ryder, 2010; Wiesmeier et al., 2016), LAD-I Syndrome (Leukocyte Adhesion Deficiency Type I; Moutsopoulos et al., 2014) or Papillon-Lefevre syndrome (Eick et al., 2014), which are associated with periodontal phenotype. On the other hand, neutrophilia, which is the accumulation of neutrophils and their histotoxic cargo in the tissue, has been clinically linked to cystic fibrosis and meningitis and may also account for the currently unknown etiology of inflammatory bowel disease, Crohn's disease, ulcerative colitis, or periodontal disease (Hajishengallis et al., 2008a; Koedel et al., 2009; Levine and Segal, 2013; Gifford and Chalmers, 2014; Prat et al., 2014). Thus, tight control of neutrophil functions and survival after bacterial clearance by induction of apoptosis, immune tolerance and resolution of inflammation, exert protective effects toward the host tissues against inflammatory tissue damage. A failure to properly regulate neutrophil abundance and turnover directly contributes to the pathogenesis of periodontitis and current literature implicates neutrophils as the main immune cell type responsible for periodontal disease progression (Hajishengallis et al., 2008b, 2015a; Scott and Krauss, 2012).

A hyper-reactive neutrophil phenotype is considered to play a central function in the periodontal disease (Chapple and Matthews, 2007; Scott and Krauss, 2012; Hajishengallis and Lamont, 2016) and, importantly, many systemic inflammatory diseases are associated with periodontitis, including cardiovascular disease, rheumatoid arthritis and diabetes type 2. As such, “hyper-inflammation” may represent one casual mechanism by which periodontitis contributes to co-morbidity (Hatanaka et al., 2006; Yasunari et al., 2006; Koziel et al., 2014b).

## Periodontitis patient-derived neutrophils

A chronic type of the periodontal disease is frequently associated with *P. gingivalis* infection (Guentsch et al., 2009). As neutrophil influx into periodontal pockets is strongly induced during such infections, several studies analyzed the functions and survival of periodontitis patient-derived neutrophils. Patients suffering from PD showed overly increased numbers of neutrophils in the oral cavity (Bender et al., 2006). Accordingly, one microarray study characterized neutrophil transcriptome changes at the site of inflammation (the oral cavity) and compared the gene expression in the periphery in healthy and PD subjects. The only significant changes were found in the “apoptosis” cluster (Lakschevitz et al., 2013). Another study revealed that GM-CSF-dependent neutrophil apoptosis was diminished in periodontitis biopsies, as analyzed by TUNEL staining. Moreover, accumulation of GM-CSF and TNF-α in gingival tissue sections was noted in the majority of diseased sites (Gamonal et al., 2003). Furthermore, functionality of tissue-derived and peripheral blood neutrophils from PD patients was deregulated. These PMNs produced higher baseline levels of ROS and elastase (Matthews et al., 2007; Guentsch et al., 2009; Aboodi et al., 2011; Damgaard et al., 2017) and secreted more pro-inflammatory cytokines, namely IL-8, IL-6, IL-1β, and TNF-α (Ling et al., 2015). Additional challenge of patient-derived neutrophils with *P. gingivalis* augmented the secretion of elastase and pro-inflammatory cytokines (Guentsch et al., 2009; Ling et al., 2015). Moreover, further exposure of PMNs to either *P. gingivalis-* or *E. coli*-derived LPS enhanced ROS generation and cathelicidin (LL-37) secretion. Similar hyper-activation of neutrophils was noted upon FcɤR engagement and treatment with either GM-CSF or phorbol ester (PMA; Johnstone et al., 2007; Matthews et al., 2007; Guentsch et al., 2009; Mariano et al., 2012). In contrast, reduced production of nitric oxide (NO) by peripheral PMNs from PD patients was noted (Mariano et al., 2012), which may actually contribute to *P. gingivalis* persistence in the gingival tissue. Importantly, a potential oral-systemic inflammatory activation loop (a self-driving sequence of events) was suggested to operate in patient-derived neutrophils. Analysis of serum isolated from aggressive and chronic periodontitis patients revealed elevated systemic antibodies to *P. gingivalis* (Guentsch et al., 2009), while plasma from periodontal patients was more efficient in neutrophil priming to fMLP and directly induced the oxidative burst (Dias et al., 2008). Interestingly, this excessive neutrophil activation could be abolished by antibodies neutralizing IL-8, GM-CSF, or IFN-α (Dias et al., 2011). Interestingly, elevated NETs release was noted in patients suffering from gingivitis, but no differences were observed in peripheral neutrophils isolated from periodontitis patients. However, NETs degradation by plasma was significantly reduced in periodontitis patients, what might contribute to the chronic inflammation, exaggerated inflammatory responses and tissue destruction observed in PD patients. Importantly, the authors noted that a non-surgical periodontal treatment restored full plasma capacity (Cooper et al., 2013; White P. et al., 2016). These patient data demonstrated that chronic periodontitis is associated with accumulation in the gingiva of hyper-active neutrophils, which exhibit an elevated release of pro-inflammatory cytokines, ROS, and anti-bacterial enzymes and show defects in apoptosis. Strikingly, *P. gingivalis*-derived LPS might be found in the bloodstream in over 50% of periodontitis patients, what clearly might modulate neutrophil and other white blood cells responsiveness (Deleon-Pennell et al., 2013). This deregulation of leukocyte function could modulate not only local, but also systemic inflammatory-immune responses and influence the risk and severity of periodontitis-associated systemic inflammatory diseases. Therefore, many studies have been devoted to elucidate molecular mechanisms underlying such aberrant functions and prolonged survival of neutrophils in the context of *P. gingivalis* infection.

### Immune host responses in mouse models of periodontal disease and their limitations

Keeping in mind that studies of periodontitis in humans are burdened with some limitations due to variables that are difficult to control among patients, animal models enable studying development, and progression of periodontal disease in a precise and controlled manner. Moreover, genetically modified mice allow to uncover defined molecular aspects of periodontitis for example involvement of specific immune cell types or signaling pathways in pathogenesis of periodontal disease.

The localized injection of periodontal pathogen *P. gingivalis* or its component into the palatal gingival tissue, oral gavage or ligature models are very convenient mice models of periodontitis. These models are characterized by periodontal tissue destruction as well as inflammation as measured by increased expression of inflammatory cytokines, such as IL-1β, IL-6, and TNF-α, which are typical hallmarks of periodontal disease observed in patients (Adamowicz et al., 2012; Saadi-Thiers et al., 2013; de Molon et al., 2013; Maekawa et al., 2014). Importantly, it was reported that the ligature model and injection of heat-killed *P. gingivalis* model were the most representative of periodontal disease in humans, whereas the oral gavage models were not effectively inducing periodontitis under the experimental conditions (de Molon et al., 2014). Thus, certainly some of these *in vivo* mouse models are burdened with limitations. Nevertheless, analysis of for example periodontitis induced by ligatures (either previously incubated with *P. gingivalis* or not) or oral gavage application of *P. gingivalis* strain ATCC 33277, revealed elevated serum levels of IL-1β and IL-6 (Saadi-Thiers et al., 2013). These results clearly indicated that *P. gingivalis* sustained periodontal inflammation. However, it is difficult to state which particular cells are responsible for this effects. Especially that pro-inflammatory cytokines such as IL-1β, IL-6, or TNF-α are produced by a variety of immune cell types (Arango Duque and Descoteaux, 2014).

The best mouse model, which allows analysis of specific immune cell types responsible for *P. gingivalis*-mediated inflammation is the mouse chamber model. This model involves injectable inoculation of bacteria into the lumen of a subcutaneously implanted titanium-coil chamber is better to pinpoint which particular cell types promote inflammation. Strikingly, over 95% of recruited cells to the chambers are Ly6G^+^ leukocytes (neutrophils). Therefore, reported by Maekawa and colleagues accumulation of pro-inflammatory mediators by in the fluid aspirated from chambers as measured by levels of IL-1β, IL-6, TNFα, or IL-17 was distinctly due to neutrophils activation (Maekawa et al., 2014). Thus, this chamber mouse model clearly confirmed that *P. gingivalis* triggers neutrophils inflammatory responses at the site of infection. Nevertheless, these *in vivo* results were validated using either ATRA differentiated HL-60 neutrophils or neutrophils isolated from human blood (Maekawa et al., 2014). Therefore, results obtained *in vivo* during analysis of mouse models of periodontitis need to be further verified *ex vivo* or *in vitro*.

Another possibility to overcome obstacles during analysis of actual cell types responsible for *P. gingivalis*-mediated inflammation is visualization by confocal microscopy. Adamowicz and colleagues quantified co-localization of infiltrating Ly6G^+^ neutrophils and pro-inflammatory IL-17 cytokine expression in periodontal tissues in *P*. *gingivalis*–induced periodontal bone loss model, clearly showing neutrophil contribution to periodontal inflammation (Adamowicz et al., 2012).

In order to pinpoint contribution of the specific immune cells in development and progression of periodontitis genetically modified mice deficient in leukocytes or lymphocytes were analyzed. For example it was shown that mice, which failed to develop MHC class II-reactive CD4^+^ T cells, presented with significantly reduced bone loss. Of note, analysis of mice devoid of NK T cells or CD8^+^ T cells did not protect mice from periodontitis (Baker et al., 1999). However, to our best knowledge, impact of neutrophils ablation on periodontal disease development and progression was not analyzed in any of mouse models of periodontitis. Instead, it was reported by Hajishengallis that loss of neutrophil infiltration inhibited disease development (Eskan et al., 2012). Mice deficient in Del-1, a negative regulator of neutrophil LFA-1-dependent recruitment to periodontium, exhibited enhanced neutrophil infiltration and IL-17-mediated inflammatory bone loss. In opposite, other authors described that IL-17-mediated neutrophil recruitment was critical in protection against *P. gingivalis*-induced alveolar bone loss in a mouse model (Yu et al., 2007). Along the same lines, other authors noted that neutrophil infiltration as well as CD45^+^ B lymphocytes was beneficial in mice infected with *P. gingivalis* (Settem et al., 2014). Importantly, IL-17 produced by Th17 cells themselves can drive periodontal bone loss in chronic *P. gingivalis* infections (Moutsopoulos et al., 2012). In chronic settings neutrophils themselves can also become a source of IL-17, leading to IL-17-dependent bone loss (Hajishengallis and Hajishengallis, 2014). Therefore, the complex role of leukocytes, lympohocytes, and IL-17 (Th17) in periodontitis remind to be determined.

Nevertheless, these results indicated that mouse models are certainly very useful tools in analysis of *P. gingivalis*-mediated periodontal inflammation and *in vivo* analysis contribute to development targeted therapeutics modulating inflammatory cascades regulated by complement (Hajishengallis and Lambris, 2013), GSK-3 (Adamowicz et al., 2012), regulatory T cells (Glowacki et al., 2013), and neutrophils (Eskan et al., 2012).

## Corruption of neutrophils by *Porphyromonas gingivalis*

*P. gingivalis* has developed a variety of mechanisms in order to overcome neutrophil-mediated killing and sustain inflammation, which allows colonization and contributes to tissue damage. This periodontopathogen utilizes several virulence factors that manipulate neutrophils recruitment, survival, and functions at the site of infections. This review will focus on virulence factors involved in the subversion of neutrophils, namely serine phosphatase (SerB), fimbriae, gingipains (RgpA, RgpB, and Kgp), *P. gingivalis* peptidyl arginine deiminase (PPAD), lipopolysaccharide (LPS), and its component (lipid A) (summarized in Table 1).

**Table 1:** ***P. gingivalis* virulence factors involved in the manipulation of neutrophil immune responses**.

**Virulence factor**	**Action**	**Consequences**	**References**
Ser B (serine phosphatase)	Dephosphorylation of Ser536 of NFκB subunit p65	Suppression of IL-8 production by GECs (so-called chemokine paralysis); abolished PMNs recruitment	Madianos et al., 1997; Bainbridge et al., 2010; Takeuchi et al., 2013
LPS	TLR2 activation and cross-talk with C5aR inhibits the Myd88 pathway while the Mal-PI3K pathway is induced	Abolition of the Myd88-dependent antimicrobial response and PI3K-mediated phagocytosis; activation of Mal-dependent inflammation	Murray and Wilton, 2003; Darveau et al., 2004; Liang et al., 2011; Maekawa et al., 2014; Olsen and Hajishengallis, 2016
LPS with penta-acylated lipid A	TLR4 agonist; pro-inflammatory potential; produced during low hemin availability	Increased expression of E-selectins; enhanced PMNs recruitment; impairment of immune tolerance; inhibition of PMNs apoptosis	Dixon and Darveau, 2005; Al-Qutub et al., 2006; Bainbridge et al., 2006; Reife et al., 2006; Berezow et al., 2009; Zaric et al., 2010
LPS with tetra-acylated lipid A	TLR4 antagonist; anti-inflammatory potential; produced during high hemin availability	Suppression of E-selectins expression; inhibition of PMNs recruitment	Dixon and Darveau, 2005; Al-Qutub et al., 2006; Bainbridge et al., 2006; Reife et al., 2006
Kgp (Lys-specific gingipain)	Degradation of IgG1, IgG3; TREM-1 degradation	Inhibition of opsonization and phagocytosis; Anti-inflammatory effect	Vincents et al., 2011; Guentsch et al., 2013
Rgps (Arg-specific gingipains)	Increased expression of TREM-1 and PAR2; shedding of sTREM-1	Elevated production of pro-inflammatory cytokines; pro-inflammatory effects	Lourbakos et al., 1998, 2001; Bostanci and Belibasakis, 2012a; Bostanci et al., 2013
High gingipain levels, Pg-bound enzymes	Degradation of IL-8 (72aa); proteolysis of TNF-α by Rgp	Abolished IL-8 dependent chemotaxis and ROS production by PMNs; diminished inflammation; chemokine paralysis	Darveau et al., 1998; Mikolajczyk-Pawlinska et al., 1998
Low gingipain levels, secreted enzymes	Selective cleavage of IL-8_77aa_ results in the generation of a truncated, hyper-active IL-8_69aa_ variant	Pro-inflammatory effects; intensified PMNs recruitment and respiratory burst	Dias et al., 2008
Rgps—initial stages of infection, secreted enzymes	Selective cleavage of C3 and C5 complement factors and generation of active C3a and C5a; induction of C5aR and TLR2 signaling pathways cross-talk (see above)	Induced PMNs chemotaxis; elevated production of pro-inflammatory cytokines and ROS	Wingrove et al., 1992; Popadiak et al., 2007; Guo et al., 2010
Rgps—advanced stages of infection, Pg-bound enzymes	Degradation of C3 and C5 complement factors	Anti-inflammatory effects	Popadiak et al., 2007; Guo et al., 2010
Kgps	Cleavage of C5aR; C4BP capture on the bacterial surface	Inhibited MPO release; suppression of the formation of the complement membrane attack complex	Jagels et al., 1996a,b; Potempa et al., 2008
Ruberythrin	Protection from oxidative stress	Pro-inflammatory effects; enhanced PMNs recruitment; resistance to reactive nitrogen species-mediated PMNs killing	Mydel et al., 2006
PPAD	C5a citrullination	Reduced pro-inflammatory potential of anaphylatoxin; protection from PMNs-mediated killing	Maresz et al., 2013; Bielecka et al., 2014
FimA Fimbriae	Increased IL-8 production; activation of TLR2 signaling and the CD11b-CD18 integrin pathway	Pro-inflammatory effects; intensification of PMNs chemotaxis, secretion of pro-inflammatory cytokines, and phagocytosis	Harokopakis and Hajishengallis, 2005; Sahingur et al., 2006; Wang et al., 2007

### PMNs deregulation by SerB

**Figure 2:**
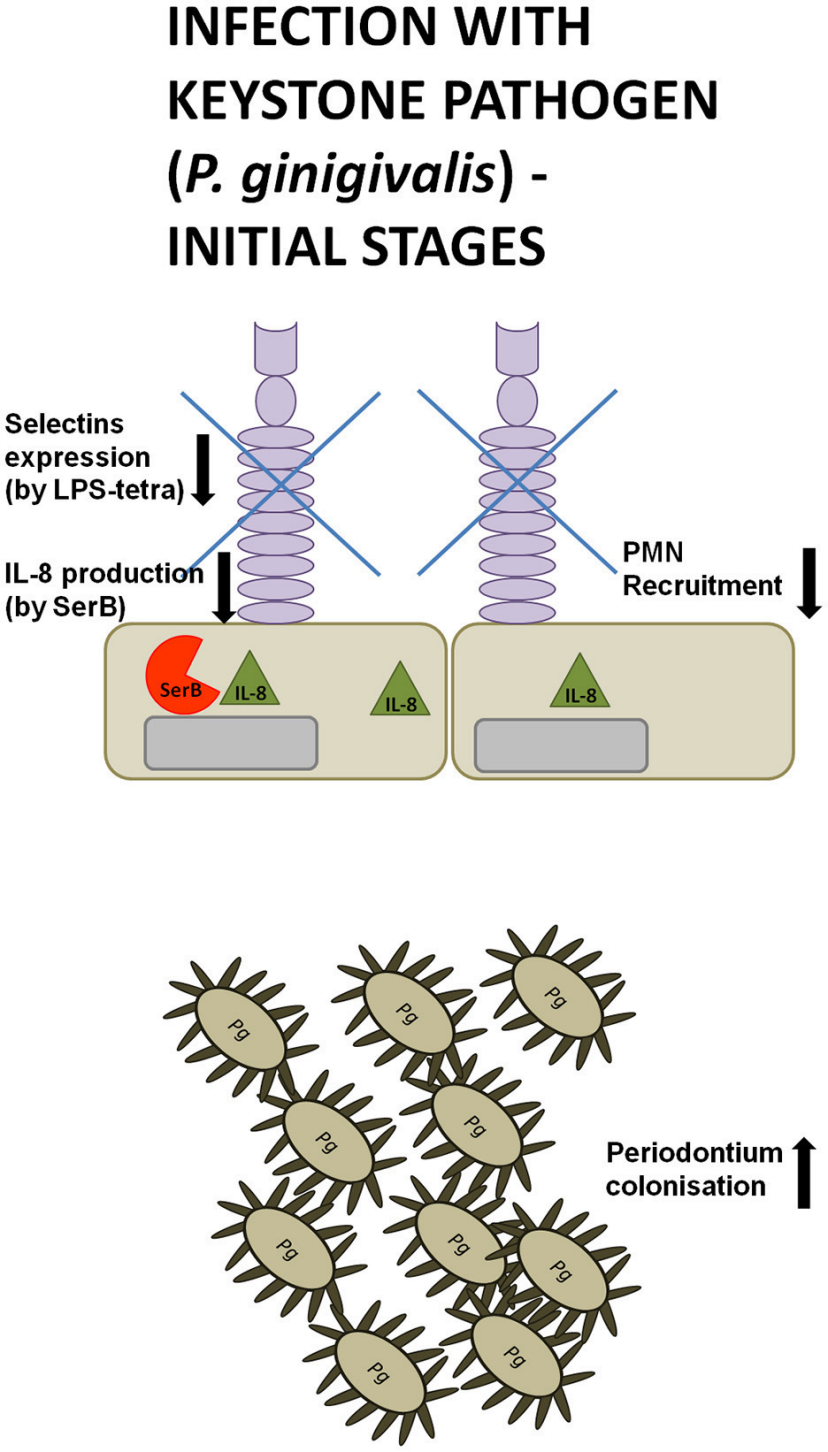
**Manipulation strategies of neutrophils and the immune system by *P*.* gingivalis* at the initial phase of infection**. During the initial phase of an infection with the keystone pathogen *P. gingivalis* (Pg) secretes serine phosphatase (SerB), inhibiting IL-8 production. At the same time, a tetra-acylated lipid A variant of *P. gingivalis* LPS suppresses the expression of L- and P-selectins on gingival epithelial cells. These manipulation strategies hinder neutrophil recruitment, giving periodontal pathogens enough time for colonization of periodontal pockets.

Serine phosphatase is an enzyme secreted by *P. gingivalis* that modulates inflammatory responses, as it was shown to inhibit IL-8 production by gingival epithelial cells through the dephosphorylation of serine S536 of p65 NF-κB subunit. This subsequently abolished PMNs recruitment and gave *P. gingivalis* enough time for colonization of periodontal pockets (Figure 2). Such insidious manipulation of cell signaling protected this periodontopathogen and bystander bacteria from neutrophil killing during the initial stages of infection. Consequently, SerB expression enhanced alveolar bone resorption in experimentally induced periodontitis in rats (Madianos et al., 1997; Bainbridge et al., 2010; Takeuchi et al., 2013). This implies that manipulation of NF-κB signaling by SerB hinders PMNs recruitment and bacterial clearance in the periodontium and therefore is involved in a pathogenic mechanism referred to as a local chemokine paralysis.

### PMNs deregulation by LPS

**Figure 3:**
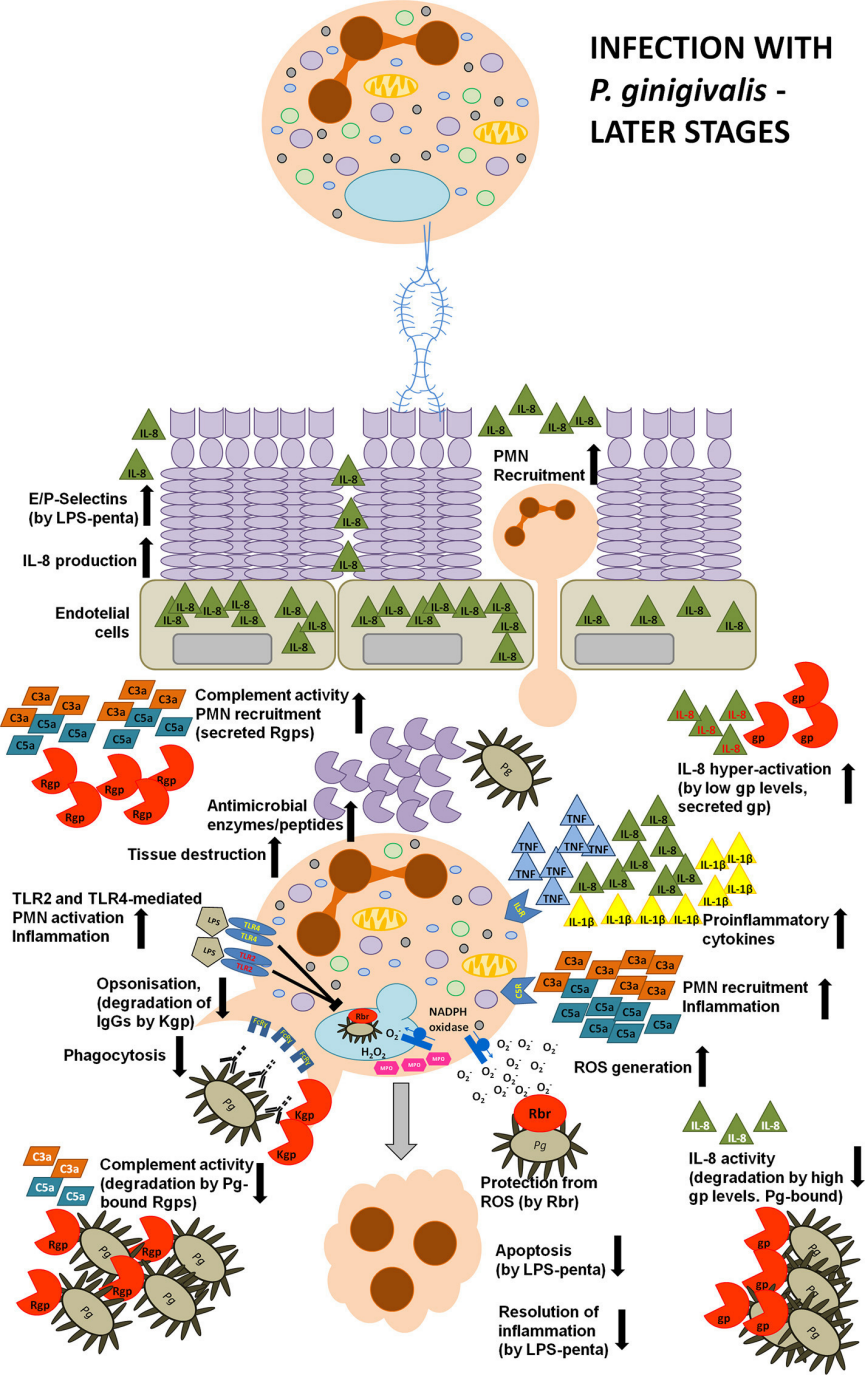
**Manipulation strategies of neutrophils and the immune system by *P*.* gingivalis* at the later phase of infection**. During a later phase of the infection *P. gingivalis* releases a penta-acylated LPS variant leading to the increased expression of L- and P-selectins on GECs and enhanced production of IL-8. This strongly stimulates neutrophil chemotaxis and transmigration to the site of infection. Moreover, P.g.-derived LPS and fimbriae strongly stimulate neutrophil pro-inflammatory and anti-bacterial responses, such as the secretion of reactive oxygen species and pro-inflammatory cytokines, and the production of anti-microbial peptides and enzymes. An elevated secretion of these anti-bacterial molecules results in gingival tissue destruction, while many virulence factors secreted by P. gingivalis protect this periodontopathogen from the consequences of hyper-inflammation. The keystone pathogen is protected from oxidative stress, as it expresses ruberythrin (Rbr) protein. Additionally, the expression of Lys-specific gingipains degrades immunoglobulins at the hinge region, and, coupled with the activation of the TLR2 signaling pathway (by LPS), abolishes bacterial opsonization and phagocytosis. Also, gingipains manipulate anti-bacterial responses by deregulating the complement cascade and IL-8-mediated neutrophil chemotaxis. In particular, depending on the concentration and the position of gingipains within the biofilm, these enzymes can exert opposing effects. C3, C5, and IL-8 are degraded at high gingipain concentration or by gingipains associated with bacterial cells or vesicles thus inhibiting pro-inflammatory responses and protecting bacteria from elimination. In contrast, low levels of soluble Arg-specific gingipains activate C5 and C3 by limited proteolysis that results in the generation of C5a and C3a anaphylatoxins. Furthermore, under such circumstances, gingipains can selectively cleave IL-8 generating a truncated, hyperactive IL-869aa variant (written in red) acting in concert with C5a and C3a in order to excessively activate neutrophil pro-inflammatory responses. Additionally, the secreted penta-acylated LPS variant diminishes neutrophil apoptosis which delays the resolution of inflammation Together, these events strongly contribute to the inflammatory tissue destruction observed in periodontitis and to excessive bleeding, providing P.g. and bystander bacteria with access to nutritional resources.

Toll-like receptors (TLR) engagement, neutrophil recruitment to the site of inflammation, pro-inflammatory cytokine release, and subsequent elimination of bacteria by PMNs are essential mechanisms of the host immune response against infection. However, after the bacterial clearance, several processes need to be engaged to prevent inflammatory tissue destruction, including the induction of immune tolerance and removal of apoptotic neutrophils from the site of infection. They contribute to the control of the host's defense and trigger resolution of inflammation (Bratton and Henson, 2011; McCracken and Allen, 2014). Lipopolysaccharide (LPS), also known as endotoxin, is a fundamental structural element of the cell envelope of gram-negative bacteria and it is able to elicit appropriate host innate responses. LPS is composed of three elements, namely O-antigen, core polysaccharide, and lipid A. The latter is responsible for the toxicity of Gram-negative bacteria and consists of di-glucosamine with two phosphate moieties at both 1′- and 4′-positions of the disaccharide backbone, where acyl chains are attached (Kumada et al., 1995; Raetz et al., 2007, 2009). However, *P. gingivalis* can deceitfully modify its lipid A structure due to both dephosphorylation and deacylation in order to manipulate host immune responses and promote chronic inflammation (Dixon and Darveau, 2005; Al-Qutub et al., 2006; Reife et al., 2006). When considering the heterogeneous acylation patterns of *P. gingivalis* lipid A, two variants are predominant, namely tetra-acylated and penta-acylated. Strikingly, these two variants were described to induce opposing host responses. The penta-acylated lipid A activated TLR4 (Toll-like receptor 4) signaling, whereas tetra-acylated lipid A variant had antagonistic effects against TLR4. Importantly, these changes in acylation were dependent on microenvironmental conditions. In particular, when bacteria were grown in low hemin conditions, LPS consisted of phosphorylated, penta-acylated lipid A structure, which exerted weak LPS agonistic effects. Whereas, during high hemin availability, mimicking an inflammatory condition, phosphorylated penta-acylated lipid A was converted into non-phosphorylated tetra-acylated lipid A, and exhibited an antagonistic activity (Al-Qutub et al., 2006). Moreover, tetra-acylated version of *P. gingivalis* lipid A was shown to inhibit E-selectin expression, while penta-acylated lipid A variant had a stimulatory effect (Reife et al., 2006). Therefore, *P. gingivalis* can exert opposing effects on the expression of this adhesion molecule expressed on endothelial cells, which impairs neutrophil transmigration during inflammation (Figures 2, 3).

On top of acylation, *P. gingivalis* can change the phosphorylation status of lipid A (Rangarajan et al., 2008). Similarly to other Gram-negative bacteria, *P. gingivalis* synthesizes lipid A that initially contains two phosphate groups at the di-glucosamine backbone. However, dephosphorylation of *P. gingivalis* lipid A can facilitate its deacylation and subsequently manipulate neutrophil responses.

It is a well-documented innate immunity paradigm that LPS is recognized by Toll-like receptor 4 of the host innate immune system, which triggers pro-inflammatory cytokine production and promotes bacteria elimination (Murdock and Nunez, 2016; Figure 1). While it is true for the LPS of most Gram-negative species, *P. gingivalis*-derived LPS is also a potent activator of TLR2 (Darveau et al., 2004). Importantly, this degenerative manipulation of TLR signaling separates pathogen phagocytosis from neutrophil inflammatory response (Figure 3). This pathologic effect was achieved by the induction of a cross-talk between the complement receptor C5aR and TLR2, which inhibited the TLR-2-MyD88 signaling pathway and activated an alternative TLR2-Mal-PI3K pathway *in vivo* (Liang et al., 2011; Maekawa et al., 2014). The authors reported that the ability to activate TLR2 signaling had a protective effect not only toward *P. gingivalis*, but also toward bystander bacteria, otherwise susceptible to bacterial killing. These studies revealed the molecular mechanism of TLR signaling manipulation by *P. gingivalis*, which clearly contributes to the persistence of dysbiotic microbial communities and drives chronic inflammation in periodontal disease (Hajishengallis and Lambris, 2011).

Neutrophil apoptosis and subsequent uptake by macrophages, referred to as efferocytosis, is among the key processes leading to the resolution of inflammation. Therefore, neutrophil resistance to cell death and diminished efferocytosis may additionally contribute to the chronic inflammation and collateral tissue damage observed in periodontal disease (Hiroi et al., 1998; Zaric et al., 2010). Indeed, neutrophils exhibited an impaired immune tolerance to *P. gingivalis* endotoxin. Stimulation with penta-acylated dephosphorylated LPS, the most potent and prevalent endotoxin isoform of *P. gingivalis*, led to continued IL-8 secretion upon secondary exposure, but inhibited TNF-α production. Interestingly, observed results were in contrast to the effect elicited by *E. coli*-derived lipopolisaccharide, which abolished the production of both cytokines upon repeated treatment (Bainbridge et al., 2006; Berezow et al., 2009). This deregulated inflammatory response to *P. gingivalis* LPS resulted in enhanced neutrophil migratory potential while apoptosis was inhibited as identified by reduced caspase-3 activity (Bainbridge and Darveau, 2001; Reife et al., 2006; Zaric et al., 2010; Figure 3). Additionally, not only LPS and lipid A, but also capsular polysaccharide isolated from three different *P. gingivalis* strains (HG-184, A7A1–28, and 381) diminished human or murine PMNs cell death in a time- and dose-dependent manner (Hiroi et al., 1998; Preshaw et al., 1999) that was not influenced by IL-10 treatment (Murray and Wilton, 2003). However, taking into account that *P. gingivalis* endotoxin may engage TLR2 instead of TLR4, this may account for its inhibitory effect on apoptosis (Bainbridge and Darveau, 2001; Murray and Wilton, 2003). This is particularly important due to the fact that *P. gingivalis* LPS can penetrate gingival tissues and consequently can exert a broad impact toward all immune cells in the periodontium and affect their responses to all bacteria present within the biofilm (Schwartz et al., 1972). Therefore, manipulation of PMN apoptosis results in an accumulation of long-lived and fully active neutrophils in the inflamed periodontium, which may contribute to the development and progression of periodontal disease.

These studies provided the molecular evidence that changes in the *P. gingivalis* endotoxin structure influence its binding affinity to TLR4. This selectively affects downstream signaling in order to evade antibacterial responses and sustain inflammation. Collectively, LPS modifications depend on the microenvironment and exert opposing actions on the host immune system. This ensures access to nutrient resources and can potentially protect the entire microbial community from TLR-mediated immune responses.

### PMNs deregulation by gingipains

Gingipains are major cysteine proteases, which are either membrane-bound or secreted and they account for 85% of the total proteolytic activity of *P. gingivalis* (Potempa et al., 1997). Based on their specificity, they are divided in two groups, namely arginine-specific (RgpA and RgpB) and lysine-specific (Kgp; Potempa et al., 2003). Working in concert, gingipains exhibit trypsin-like activity and can cleave many host components, such as the extracellular matrix, cytokines, immunoglobulins, or complement factors (Guo et al., 2010). However, these proteases are not merely responsible for an indiscriminate degradation of proteins, but show a large degree of specificity in action that accounts for much of *P. gingivalis* resistance to host antibacterial strategies (Bostanci and Belibasakis, 2012b). Gingipains are able to override host defense mechanisms, such as antibody opsonization, complement system activation, or pro-inflammatory signaling, which ensures this periodontopathogen's exceptional resistance to the bactericidal activity of the human serum and the killing potential of neutrophils (Figure 3).

Opsonization by natural or acquired antibodies is an important protective feature of innate and adaptive immune response and facilitates phagocytosis of invading bacteria. However, gingipain K (Kgp) has the unique ability to cleave IgG1 and IgG3 at the hinge region, thus separating the antigen binding Fab fragment from the effector Fc fragment of immunoglobulins. This activity was observed not only *in vitro* using isolated IgGs or human plasma, but it was also detected *in vivo* in gingival crevicular fluid (GCF). Consequently, treatment of patient serum samples with Kgp inhibited *P. gingivalis* opsonization and subsequent phagocytosis by neutrophils (Kobayashi et al., 2001; Guentsch et al., 2011; Vincents et al., 2011). Therefore, lysine-specific gingipain suppresses IgG-mediated opsonization and *P. gingivalis* phagocytosis, which contributes to pathogen persistence.

Gingipains can also influence the host innate immune response by a manipulation of signaling dependent on the TREM (Triggering Receptor Expressed on Myeloid cells) or PAR (Protease-Activated Receptors) families of cell receptors. TREM-1 receptor belongs to the immunoglobulin family and is involved in the amplification of pro-inflammatory cytokines production as well as regulation of apoptosis. It was reported that gingipains increased the expression of TREM-1 on neutrophils, which resulted in an elevated production of IL-8. Interestingly, depending on gingipain specificity, authors noted opposing biological effects. Arg-specific gingipains were capable of shedding a soluble TREM-1 (sTREM-1) from the PMNs surface, while Lys-specific gingipains were degrading TREM-1 (Bostanci and Belibasakis, 2012a; Bostanci et al., 2013).

Gingipains were also shown to deregulate PAR signaling in neutrophils, gingival fibroblasts, epithelial cells, and T lymphocytes. In particular, Rgps were able to activate PAR2, a G-protein-coupled transmembrane receptor expressed, among others, on neutrophils. Rgps-mediated PAR2 activation enhanced the biosynthesis of prostaglandin E2, IFN-ɤ, IL-1β, and IL-6, which led to accelerated alveolar bone loss in experimentally-induced periodontitis in mice. Therefore, induction of PAR expression by gingipains is designed to sustain inflammation in *P. gingivalis*-infected periodontium (Lourbakos et al., 1998, 2001; Bostanci and Belibasakis, 2012b). Importantly, authors also reported an elevated expression of PAR2 on neutrophils from periodontitis patients, which indicated that deregulated PAR2 expression may contribute to periodontal inflammation severity (Holzhausen et al., 2006, 2010). Therefore, differential regulation of TREM-1 or PAR2 signaling by gingipains is designed to manipulate the host innate immune responses and contributes to chronic periodontal inflammation.

Additionally, gingipains were also shown to exploit IL-8 signaling in order to sustain inflammation, deregulate neutrophil chemotaxis and eventually inhibit bacterial elimination. Interleukin 8 (IL-8) is a chemokine known as a neutrophil chemotactic factor orchestrating many PMNs bactericidal activities, such as phagocytosis, respiratory burst, production of anti-bacterial enzymes, and pro-inflammatory cytokines. *P. gingivalis* LPS is able to induce the secretion of IL-8_72aa_ and IL-8_77aa_ variants from immune and non-immune cells, respectively. While the first variant is very potent in priming neutrophils, the latter needs to be truncated on the N-terminus to elicit full activity. This functional and structural diversity of IL-8 is exploited by *P. gingivalis*, which can manipulate IL-8 signaling depending on the stage of infection, gingipain concentration as well as the proximity of the cytokine to the bacterial plaque (Figure 3). Specifically, Arg- or Lys-gingipains can diminish IL-8_72aa_ activity and inhibit neutrophil chemotaxis as well as ROS production induced by fMLP during initial stages of infection, thus significantly contributing to a local chemokine paralysis. On the contrary, during later stages of *P. gingivalis* infection, the limited proteolysis of IL-8_77aa_ by R- and K-gingipains in the presence of fMLP resulted in the generation of truncated hyperactive IL-8_69aa_ variant, which enhanced PMNs recruitment and oxygen-dependent bacterial elimination (Darveau et al., 1998; Dias et al., 2008). Additionally, it was shown that enzymes associated with the bacterial outer-membrane blebs (vesicles) degrade and inactivate this chemokine, whereas soluble gingipains were able to process IL-8_77aa_ into the hyper-active variant (Mikolajczyk-Pawlinska et al., 1998). Of note, Arg-specific gingipains interfere also with TNF-α signaling by degrading the soluble as well as membrane-bound forms of this cytokine (Mezyk-Kopec et al., 2005), which attenuated host immune response and neutrophil recruitment, both especially important during initial stages of *P. gingivalis* infection.

Also, various complement components strongly regulate PMNs activity as these cells constitutively express several complement receptors, such as CR1 (CD35), CR3 (CD11b/CD18, Mac-1), CR4 (CD11c/CD18, Mac-1), or CR5 receptors. Engagement of complement receptors activates many different signaling pathways and triggers neutrophil migration, phagocytosis, degranulation, intracellular killing, or ROS production (Krauss et al., 2010; Hajishengallis et al., 2015b). Not surprisingly, another strategy of immune evasion employed by *P. gingivalis* is the inactivation of complement factors (Figure 3). This periodontopathogen produces high quantities of gingipains targeting these fundamental complement-mediated innate immune responses (Popadiak et al., 2007). In particular, RgpA can precisely cleave C3 and C5 to produce active C3b and C5a, the latter being an anaphilatixin, strongly promoting neutrophil recruitment to the gingiva (Wingrove et al., 1992). Actually, gingipains have been shown to have complex, “biphasic” effects on the complement system. At low concentrations of gingipains, which mimic early infection stages, they precisely cleave C3 and C5 and generate active C3a and C5a, respectively. In turn, at high concentrations or at deeper gingival localization, where the biofilm resides, they inhibit the complement pathway. This indicates that at the beginning of bacterial invasion, gingipains enhance inflammation in order to increase nutrients supply, while at advanced stages of periodontal disease they inactivate the complement cascade, which is designed to protect *P. gingivalis* and bystander bacteria from the bactericidal activity of the human serum and neutrophil killing (Popadiak et al., 2007; Potempa and Pike, 2009).

Moreover, Lys-specific gingipain was shown to block C5a-mediated anti-microbial activity by cleaving C5aR and abolishing myeloperoxidase release by neutrophils (Jagels et al., 1996a,b). Interestingly, membrane-bound RgpA can capture complement inhibitor C4BP on *P. gingivalis* surface, which abolishes membrane complement attack (MAC; Potempa et al., 2008). Furthermore, gingipains were also shown to interfere with the clotting cascade by degradation of fibrinogen. Especially Kgp increased bleeding tendency at the site of infection and prolonged inflammation (Imamura et al., 1995, 1997; Rapala-Kozik et al., 2011).

This data proved that *P. gingivalis* represents a periodontal pathogen, which employs different strategies to gain access to nutrient resources and overcome neutrophil-mediated killing. Not only endogenous host enzymes, but also gingipains mediate periodontal tissue breakdown. Moreover, gingipains, which are found at very high concentrations in periodontal pockets, deregulate the complement cascade, opsonization by immunoglobulins and receptor signaling, collectively leading to increased vascular permeability and bleeding. These effects increase the availability of hemin required for bacterial growth and facilitate the spreading of the pathogen. Depending on the stage of infection, position within the biofilm and specific circumstances, this periodontopathogen can either promote or prevent such activities as complement activation, neutrophil recruitment and inflammatory response. However, observed *in vitro* suppression of inflammatory responses, which manipulate neutrophils infiltration to the periodontium at the very early stages of *P. gingivalis* colonization was not observed *in vivo* during analysis of mouse models of periodontitis. However, it is difficult to establish *in vivo* relevant *in vitro* systems as either cell lines or isolated primary neutrophils are separated from their normal environment. On one hand, *in vitro* approaches allow to study molecular mechanisms in a very precise and controlled manner. On the other hand, *in vitro* work simplifies the system under study. Therefore, conclusions based on *in vitro* approaches or mouse models will be ultimately verified by clinical trials involving periodontitis patients.

Collectively, this compartmentalization of pro- and anti-inflammatory responses protects *P. gingivalis* from elimination and fuels the inflammation in distal positions from bacterial plaque, which clearly contributes to inflammatory tissue damage observed in patients suffering from the periodontal disease.

### PMNs deregulation by RBR

*P. gingivalis* expresses a non-haem iron protein called rubrerythrin (Rbr) in order to avoid oxidative stress and gain protection from reactive oxygen and nitrogen species. Rbr-positive *P. gingivalis* was shown to be more resistant to neutrophil killing, which enabled colonization of the periodontal tissues. Moreover, this virulence factor increased the mobilization and activation of neutrophils which was indispensable for the establishment of inflammation and contributed to greater tissue damage *in vivo* (Mydel et al., 2006). These results suggest that the host respiratory burst is manipulated by rubrerythrin in order to provoke inflammation which, paradoxically, promotes *P. gingivalis* survival.

### PMNs deregulation by PPAD

*P. gingivalis* peptidyl arginine deiminase (PPAD) is another enzyme unique to *P. gingivalis*, which converts proteins Arg residues, usually present at the carboxy terminus, into citrulline (McGraw et al., 1999). This modification is implicated in the development of rheumatoid arthritis. However, it was also shown that C5a citrullination reduced the chemotactic and proinflammatory potential of this anaphylatoxin (Maresz et al., 2013; Bielecka et al., 2014; Koziel et al., 2014a). Therefore, modifications of host proteins by PPAD represent another manipulative strategy designed to protect *P. gingivalis* and bystander bacteria from neutrophil-mediated killing.

### PMNs deregulation by fimbriae

Fimbriae, also called pili, are proteinaceous appendages on the bacterial outer surface, which promote both adhesion to and invasion of the host cells (Enersen et al., 2013). In order to colonize the subgingival region, *P. gingivalis* utilizes two types of fimbria, namely long, composed of the FimA protein, and short, or minor, which consists of the Mfa protein. However, fimbriae do not only facilitate bacterial invasion, but also manipulate neutrophil responses. Challenge of PMNs with FimA, a main structural protein of the *P. gingivalis* long fimbriae, resulted in an increased release of the pro-inflammatory cytokine IL-8 and enhanced fibrinogen binding, which could be further augmented by additional stimulation with fMLP (Sahingur et al., 2006). Detailed analysis of neutrophil signaling revealed that *P. gingivalis* fimbriae activated TLR2 signaling by employing CD14 and PI3K. This, in turn, induced activation in neutrophils of CD11b-CD18 (also called Mac-1 or CR3) integrins (Harokopakis and Hajishengallis, 2005), which are the most abundant integrin type. Usually, Mac-1 engagement trigger inside-out signaling, resulting in inflammatory and antibacterial responses, such as leukocyte adhesion, transmigration, production of inflammatory cytokines, and phagocytosis (Ehlers, 2000). However, activation of CR3 by *P. gingivalis* was reported to facilitate the invasion and colonization of macrophages, which subsequently served as an intracellular bacterial reservoir. Therefore, it would be interesting to investigate whether similar mechanisms are employed to corrupt PMNs. Collectively, already published results undoubtedly show that *P. gingivalis* fimbriae strongly manipulate neutrophil signaling in order to prolong a nutritionally favorable inflammatory response and promote bacterial persistence.

## Conclusions and future directions

Neutrophil homeostasis in the periodontium is ensured by a balance between neutrophil migration to the site of infection, anti-bacterial and pro-inflammatory response, and finally the clearance of apoptotic PMNs during the resolution of inflammation. However, excessive neutrophil recruitment to the periodontium and their hyper-activation arises as a novel mechanism strongly contributing to the development and progression of periodontal disease. Different *P. gingivalis* virulence factors corrupt many neutrophil functions in order to sustain inflammation, gain access to nutrient resources and ensure protection from killing. This suggests that neutrophil contribution to the progression of periodontitis might not only be related to defective elimination of bacteria, but additionally involve a deregulation of immune tolerance, neutrophil apoptosis and mechanisms driving resolution of inflammation. However, depending on the specific circumstances and the stage of bacterial infection, the manipulation of PMNs might actually lead to opposing effects, either pro- or anti-inflammatory. Therefore, future research might discover the link between regulatory defects in anti-bacterial or effector neutrophil functions and the progression of chronic periodontal disease. A better molecular understanding of periodontitis-associated neutrophil dysfunctions due to their manipulation by *P. gingivalis* could be used as a potential targeted therapy in patients.

## Author contributions

MS wrote the manuscript, prepared figures, and the table; JP corrected the manuscript.

## Funding

This work was supported by funding grants Foundation for Polish Science (Homing/2016-1/9) to MS, US NIH/NIDCR (DE 022597 and DE 023207), and National Science Center (2012/04/A/NZ1/00051, NCN, Krakow, Poland) to JP.

### Conflict of interest statement

The authors declare that the research was conducted in the absence of any commercial or financial relationships that could be construed as a potential conflict of interest.

## References

[B1] Aboodi G. M. Goldberg M. B. Glogauer M. (2011). Refractory periodontitis population characterized by a hyperactive oral neutrophil phenotype. J. Periodontol. 82, 726–733. 10.1902/jop.2010.100508 21080789

[B2] Adamowicz K. Wang H. Jotwani R. Zeller I. Potempa J. Scott D. A. (2012). Inhibition of GSK3 abolishes bacterial-induced periodontal bone loss in mice. Mol. Med. 18, 1190–1196. 10.2119/molmed.2012.00180 22847803 PMC3510296

[B3] Al-Qutub M. N. Braham P. H. Karimi-Naser L. M. Liu X. Genco C. A. Darveau R. P. (2006). Hemin-dependent modulation of the lipid A structure of *Porphyromonas gingivalis* lipopolysaccharide. Infect. Immun. 74, 4474–4485. 10.1128/IAI.01924-05 16861633 PMC1539574

[B4] Arango Duque G. Descoteaux A. (2014). Macrophage cytokines: involvement in immunity and infectious diseases. Front. Immunol. 5:491. 10.3389/fimmu.2014.00491 25339958 PMC4188125

[B5] Bainbridge B. W. Darveau R. P. (2001). *Porphyromonas gingivalis* lipopolysaccharide: an unusual pattern recognition receptor ligand for the innate host defense system. Acta Odontol. Scand. 59, 131–138. 10.1080/000163501750266710 11501881

[B6] Bainbridge B. W. Coats S. R. Pham T. T. Reife R. A. Darveau R. P. (2006). Expression of a *Porphyromonas gingivalis* lipid A palmitylacyltransferase in *Escherichia coli* yields a chimeric lipid A with altered ability to stimulate interleukin-8 secretion. Cell. Microbiol. 8, 120–129. 10.1111/j.1462-5822.2005.00605.x 16367871

[B7] Bainbridge B. Verma R. K. Eastman C. Yehia B. Rivera M. Moffatt C.. (2010). Role of *Porphyromonas gingivalis* phosphoserine phosphatase enzyme SerB in inflammation, immune response, and induction of alveolar bone resorption in rats. Infect. Immun. 78, 4560–4569. 10.1128/IAI.00703-10 20805334 PMC2976320

[B8] Baker P. J. Dixon M. Evans R. T. Dufour L. Johnson E. Roopenian D. C. (1999). CD4(+) T cells and the proinflammatory cytokines gamma interferon and interleukin-6 contribute to alveolar bone loss in mice. Infect. Immun. 67, 2804–2809. 10338484 10.1128/iai.67.6.2804-2809.1999 PMC96585

[B9] Bender J. S. Thang H. Glogauer M. (2006). Novel rinse assay for the quantification of oral neutrophils and the monitoring of chronic periodontal disease. J. Periodont. Res. 41, 214–220. 10.1111/j.1600-0765.2005.00861.x 16677291

[B10] Berezow A. B. Ernst R. K. Coats S. R. Braham P. H. Karimi-Naser L. M. Darveau R. P. (2009). The structurally similar, penta-acylated lipopolysaccharides of *Porphyromonas gingivalis* and Bacteroides elicit strikingly different innate immune responses. Microb. Pathog. 47, 68–77. 10.1016/j.micpath.2009.04.015 19460428 PMC2707506

[B11] Bielecka E. Scavenius C. Kantyka T. Jusko M. Mizgalska D. Szmigielski B.. (2014). Peptidyl arginine deiminase from *Porphyromonas gingivalis* abolishes anaphylatoxin C5a activity. J. Biol. Chem. 289, 32481–32487. 10.1074/jbc.C114.617142 25324545 PMC4239603

[B12] Borregaard N. (2010). Neutrophils, from marrow to microbes. Immunity 33, 657–670. 10.1016/j.immuni.2010.11.011 21094463

[B13] Borregaard N. Herlin T. (1982). Energy metabolism of human neutrophils during phagocytosis. J. Clin. Invest. 70, 550–557. 10.1172/JCI110647 7107894 PMC370256

[B14] Bostanci N. Belibasakis G. N. (2012a). Doxycycline inhibits TREM-1 induction by *Porphyromonas gingivalis*. FEMS Immunol. Med. Microbiol. 66, 37–44. 10.1111/j.1574-695X.2012.00982.x 22540741

[B15] Bostanci N. Belibasakis G. N. (2012b). *Porphyromonas gingivalis*: an invasive and evasive opportunistic oral pathogen. FEMS Microbiol. Lett. 333, 1–9. 10.1111/j.1574-695X.2012.00982.x 22530835

[B16] Bostanci N. Thurnheer T. Aduse-Opoku J. Curtis M. A. Zinkernagel A. S. Belibasakis G. N. (2013). *Porphyromonas gingivalis* regulates TREM-1 in human polymorphonuclear neutrophils via its gingipains. PLoS ONE 8:e75784. 10.1371/journal.pone.0075784 24124513 PMC3790815

[B17] Bratton D. L. Henson P. M. (2011). Neutrophil clearance: when the party is over, clean-up begins. Trends Immunol. 32, 350–357. 10.1016/j.it.2011.04.009 21782511 PMC3151332

[B18] Chapple I. L. Matthews J. B. (2007). The role of reactive oxygen and antioxidant species in periodontal tissue destruction. Periodontol. 2000 43, 160–232. 10.1111/j.1600-0757.2006.00178.x 17214840

[B19] Cooper P. R. Palmer L. J. Chapple I. L. (2013). Neutrophil extracellular traps as a new paradigm in innate immunity: friend or foe? Periodontol. 2000 63, 165–197. 10.1111/prd.12025 23931060

[B20] Czabotar P. E. Lessene G. Strasser A. Adams J. M. (2014). Control of apoptosis by the BCL-2 protein family: implications for physiology and therapy. Nat. Rev. Mol. Cell Biol. 15, 49–63. 10.1038/nrm3722 24355989

[B21] Damgaard C. Kantarci A. Holmstrup P. Hasturk H. Nielsen C. H. Van Dyke T. E. (2017). *Porphyromonas gingivalis*-induced production of reactive oxygen species, tumor necrosis factor-alpha, interleukin-6, CXCL8 and CCL2 by neutrophils from localized aggressive periodontitis and healthy donors: modulating actions of red blood cells and resolvin E1. J. Periodontal. Res. 52, 246–254. 10.1111/jre.12388 27146665 PMC5097708

[B22] Darveau R. P. Belton C. M. Reife R. A. Lamont R. J. (1998). Local chemokine paralysis, a novel pathogenic mechanism for *Porphyromonas gingivalis*. Infect. Immun. 66, 1660–1665. 9529095 10.1128/iai.66.4.1660-1665.1998 PMC108102

[B23] Darveau R. P. Hajishengallis G. Curtis M. A. (2012). *Porphyromonas gingivalis* as a potential community activist for disease. J. Dent. Res. 91, 816–820. 10.1177/0022034512453589 22772362 PMC3420389

[B24] Darveau R. P. Pham T. T. Lemley K. Reife R. A. Bainbridge B. W. Coats S. R.. (2004). *Porphyromonas gingivalis* lipopolysaccharide contains multiple lipid A species that functionally interact with both toll-like receptors 2 and 4. Infect. Immun. 72, 5041–5051. 10.1128/IAI.72.9.5041-5051.2004 15321997 PMC517442

[B25] de Molon R. S. de Avila E. D. Cirelli J. A. (2013). Host responses induced by different animal models of periodontal disease: a literature review. J. Investig. Clin. Dent. 4, 211–218. 10.1111/jicd.12018 23188588

[B26] de Molon R. S. de Avila E. D. Boas Nogueira A. V. Chaves de Souza J. A. Avila-Campos M. J. de Andrade C. R.. (2014). Evaluation of the host response in various models of induced periodontal disease in mice. J. Periodontol. 85, 465–477. 10.1902/jop.2013.130225 23805811

[B27] Deleon-Pennell K. Y. de Castro Bras L. E. Lindsey M. L. (2013). Circulating *Porphyromonas gingivalis* lipopolysaccharide resets cardiac homeostasis in mice through a matrix metalloproteinase-9-dependent mechanism. Physiol. Rep. 1:e00079. 10.1002/phy2.79 24159380 PMC3804276

[B28] Dias I. H. Marshall L. Lambert P. A. Chapple I. L. Matthews J. B. Griffiths H. R. (2008). Gingipains from *Porphyromonas gingivalis* increase the chemotactic and respiratory burst-priming properties of the 77-amino-acid interleukin-8 variant. Infect. Immun. 76, 317–323. 10.1128/IAI.00618-07 18025101 PMC2223636

[B29] Dias I. H. Matthews J. B. Chapple I. L. Wright H. J. Dunston C. R. Griffiths H. R. (2011). Activation of the neutrophil respiratory burst by plasma from periodontitis patients is mediated by pro-inflammatory cytokines. J. Clin. Periodontol. 38, 1–7. 10.1111/j.1600-051X.2010.01628.x 20964702

[B30] Dixon D. R. Darveau R. P. (2005). Lipopolysaccharide heterogeneity: innate host responses to bacterial modification of lipid a structure. J. Dent. Res. 84, 584–595. 10.1177/154405910508400702 15972584

[B31] Ehlers M. R. (2000). CR3: a general purpose adhesion-recognition receptor essential for innate immunity. Microbes Infect. 2, 289–294. 10.1016/S1286-4579(00)00299-9 10758405

[B32] Eick S. Puklo M. Adamowicz K. Kantyka T. Hiemstra P. Stennicke H.. (2014). Lack of cathelicidin processing in Papillon-Lefevre syndrome patients reveals essential role of LL-37 in periodontal homeostasis. Orphanet J. Rare Dis. 9:148. 10.1186/s13023-014-0148-y 25260376 PMC4181722

[B33] Enersen M. Nakano K. Amano A. (2013). *Porphyromonas gingivalis* fimbriae. J. Oral Microbiol. 5:20265. 10.3402/jom.v5i0.20265 23667717 PMC3647041

[B34] Eskan M. A. Jotwani R. Abe T. Chmelar J. Lim J. H. Liang S.. (2012). The leukocyte integrin antagonist Del-1 inhibits IL-17-mediated inflammatory bone loss. Nat. Immunol. 13, 465–473. 10.1038/ni.2260 22447028 PMC3330141

[B35] Gamonal J. Sanz M. O'Connor A. Acevedo A. Suarez I. Sanz A.. (2003). Delayed neutrophil apoptosis in chronic periodontitis patients. J. Clin. Periodontol. 30, 616–623. 10.1034/j.1600-051X.2003.00350.x 12834499

[B36] Garcia Saez A. J. Villunger A. (2016). MOMP in the absence of BH3-only proteins. Genes Dev. 30, 878–880. 10.1101/gad.281519.116 27083995 PMC4840294

[B37] Gifford A. M. Chalmers J. D. (2014). The role of neutrophils in cystic fibrosis. Curr. Opin. Hematol. 21, 16–22. 10.1097/MOH.0000000000000009 24253427

[B38] Glowacki A. J. Yoshizawa S. Jhunjhunwala S. Vieira A. E. Garlet G. P. Sfeir C.. (2013). Prevention of inflammation-mediated bone loss in murine and canine periodontal disease via recruitment of regulatory lymphocytes. Proc. Natl. Acad. Sci. U.S.A. 110, 18525–18530. 10.1073/pnas.1302829110 24167272 PMC3831997

[B39] Graves D. T. Fine D. Teng Y. T. Van Dyke T. E. Hajishengallis G. (2008). The use of rodent models to investigate host-bacteria interactions related to periodontal diseases. J. Clin. Periodontol. 35, 89–105. 10.1111/j.1600-051X.2007.01172.x 18199146 PMC2649707

[B40] Guentsch A. Hirsch C. Pfister W. Vincents B. Abrahamson M. Sroka A.. (2013). Cleavage of IgG1 in gingival crevicular fluid is associated with the presence of *Porphyromonas gingivalis*. J. Periodont. Res. 48, 458–465. 10.1111/jre.12027 23116446 PMC3566341

[B41] Guentsch A. Kramesberger M. Sroka A. Pfister W. Potempa J. Eick S. (2011). Comparison of gingival crevicular fluid sampling methods in patients with severe chronic periodontitis. J. Periodontol. 82, 1051–1060. 10.1902/jop.2011.100565 21235330 PMC3129431

[B42] Guentsch A. Puklo M. Preshaw P. M. Glockmann E. Pfister W. Potempa J.. (2009). Neutrophils in chronic and aggressive periodontitis in interaction with *Porphyromonas gingivalis* and Aggregatibacter actinomycetemcomitans. J. Periodont. Res. 44, 368–377. 10.1111/j.1600-0765.2008.01113.x 19210340 PMC4180098

[B43] Guo Y. Nguyen K. A. Potempa J. (2010). Dichotomy of gingipains action as virulence factors: from cleaving substrates with the precision of a surgeon's knife to a meat chopper-like brutal degradation of proteins. Periodontol. 2000 54, 15–44. 10.1111/j.1600-0757.2010.00377.x 20712631 PMC2924770

[B44] Hajishengallis E. Hajishengallis G. (2014). Neutrophil homeostasis and periodontal health in children and adults. J. Dent. Res. 93, 231–237. 10.1177/0022034513507956 24097856 PMC3929973

[B45] Hajishengallis G. (2014). Immunomicrobial pathogenesis of periodontitis: keystones, pathobionts, and host response. Trends Immunol. 35, 3–11. 10.1016/j.it.2013.09.001 24269668 PMC3947349

[B46] Hajishengallis G. Lambris J. D. (2011). Microbial manipulation of receptor crosstalk in innate immunity. Nat. Rev. Immunol. 11, 187–200. 10.1038/nri2918 21350579 PMC3077082

[B47] Hajishengallis G. Lambris J. D. (2013). Complement-targeted therapeutics in periodontitis. Adv. Exp. Med. Biol. 735, 197–206. 10.1007/978-1-4614-4118-2_13 23402028 PMC3466049

[B48] Hajishengallis G. Lamont R. J. (2012). Beyond the red complex and into more complexity: the polymicrobial synergy and dysbiosis (PSD) model of periodontal disease etiology. Mol. Oral Microbiol. 27, 409–419. 10.1111/j.2041-1014.2012.00663.x 23134607 PMC3653317

[B49] Hajishengallis G. Lamont R. J. (2016). Dancing with the stars: how choreographed bacterial interactions dictate nososymbiocity and give rise to keystone pathogens, accessory pathogens, and pathobionts. Trends Microbiol. 24, 477–489. 10.1016/j.tim.2016.02.010 26968354 PMC4874887

[B50] Hajishengallis G. Chavakis T. Hajishengallis E. Lambris J. D. (2015a). Neutrophil homeostasis and inflammation: novel paradigms from studying periodontitis. J. Leukoc. Biol. 98, 539–548. 10.1189/jlb.3VMR1014-468R 25548253 PMC4569046

[B51] Hajishengallis G. Maekawa T. Abe T. Hajishengallis E. Lambris J. D. (2015b). Complement involvement in periodontitis: molecular mechanisms and rational therapeutic approaches. Adv. Exp. Med. Biol. 865, 57–74. 10.1007/978-3-319-18603-0_4 26306443 PMC4562417

[B52] Hajishengallis G. Wang M. Bagby G. J. Nelson S. (2008a). Importance of TLR2 in early innate immune response to acute pulmonary infection with *Porphyromonas gingivalis* in mice. J. Immunol. 181, 4141–4149. 10.4049/jimmunol.181.6.4141 18768871 PMC2625304

[B53] Hajishengallis G. Wang M. Liang S. Shakhatreh M. A. James D. Nishiyama S.. (2008b). Subversion of innate immunity by periodontopathic bacteria via exploitation of complement receptor-3. Adv. Exp. Med. Biol. 632, 203–219. 10.1007/978-0-387-78952-1_15 19025124 PMC2649712

[B54] Harokopakis E. Hajishengallis G. (2005). Integrin activation by bacterial fimbriae through a pathway involving CD14, Toll-like receptor 2, and phosphatidylinositol-3-kinase. Eur. J. Immunol. 35, 1201–1210. 10.1002/eji.200425883 15739163

[B55] Hatanaka E. Monteagudo P. T. Marrocos M. S. Campa A. (2006). Neutrophils and monocytes as potentially important sources of proinflammatory cytokines in diabetes. Clin. Exp. Immunol. 146, 443–447. 10.1111/j.1365-2249.2006.03229.x 17100763 PMC1810405

[B56] Hiroi M. Shimojima T. Kashimata M. Miyata T. Takano H. Takahama M.. (1998). Inhibition by *Porphyromonas gingivalis* LPS of apoptosis induction in human peripheral blood polymorphonuclear leukocytes. Anticancer Res. 18, 3475–3479. 9858927

[B57] Holzhausen M. Cortelli J. R. da Silva V. A. Franco G. C. Cortelli S. C. Vergnolle N. (2010). Protease-activated receptor-2 (PAR(2)) in human periodontitis. J. Dent. Res. 89, 948–953. 10.1177/0022034510373765 20530726

[B58] Holzhausen M. Spolidorio L. C. Ellen R. P. Jobin M. C. Steinhoff M. Andrade-Gordon P.. (2006). Protease-activated receptor-2 activation: a major role in the pathogenesis of *Porphyromonas gingivalis* infection. Am. J. Pathol. 168, 1189–1199. 10.2353/ajpath.2006.050658 16565494 PMC1606564

[B59] Houle M. A. Grenier D. Plamondon P. Nakayama K. (2003). The collagenase activity of *Porphyromonas gingivalis* is due to Arg-gingipain. FEMS Microbiol. Lett. 221, 181–185. 10.1016/S0378-1097(03)00178-2 12725924

[B60] Imamura T. Potempa J. Pike R. N. Moore J. N. Barton M. H. Travis J. (1995). Effect of free and vesicle-bound cysteine proteinases of *Porphyromonas gingivalis* on plasma clot formation: implications for bleeding tendency at periodontitis sites. Infect. Immun. 63, 4877–4882. 7591149 10.1128/iai.63.12.4877-4882.1995 PMC173698

[B61] Imamura T. Potempa J. Tanase S. Travis J. (1997). Activation of blood coagulation factor X by arginine-specific cysteine proteinases (gingipain-Rs) from *Porphyromonas gingivalis*. J. Biol. Chem. 272, 16062–16067. 10.1074/jbc.272.25.16062 9188512

[B62] Jagels M. A. Ember J. A. Travis J. Potempa J. Pike R. Hugli T. E. (1996a). Cleavage of the human C5A receptor by proteinases derived from *Porphyromonas gingivalis*: cleavage of leukocyte C5a receptor. Adv. Exp. Med. Biol. 389, 155–164. 10.1007/978-1-4613-0335-0_19 8861006

[B63] Jagels M. A. Travis J. Potempa J. Pike R. Hugli T. E. (1996b). Proteolytic inactivation of the leukocyte C5a receptor by proteinases derived from *Porphyromonas gingivalis*. Infect. Immun. 64, 1984–1991. 8675297 10.1128/iai.64.6.1984-1991.1996 PMC174026

[B64] Johnstone A. M. Koh A. Goldberg M. B. Glogauer M. (2007). A hyperactive neutrophil phenotype in patients with refractory periodontitis. J. Periodontol. 78, 1788–1794. 10.1902/jop.2007.070107 17760550

[B65] Kassebaum N. J. Bernabe E. Dahiya M. Bhandari B. Murray C. J. Marcenes W. (2014). Global burden of severe periodontitis in 1990-2010: a systematic review and meta-regression. J. Dent. Res. 93, 1045–1053. 10.1177/0022034514552491 25261053 PMC4293771

[B66] Kobayashi T. Yamamoto K. Sugita N. van Spriel A. B. Kaneko S. van de Winkel J. G.. (2001). Effective *in vitro* clearance of *Porphyromonas gingivalis* by Fc alpha receptor I (CD89) on gingival crevicular neutrophils. Infect. Immun. 69, 2935–2942. 10.1128/IAI.69.5.2935-2942.2001 11292709 PMC98245

[B67] Koedel U. Frankenberg T. Kirschnek S. Obermaier B. Hacker H. Paul R.. (2009). Apoptosis is essential for neutrophil functional shutdown and determines tissue damage in experimental pneumococcal meningitis. PLoS Pathog. 5:e1000461. 10.1371/journal.ppat.1000461 19478887 PMC2682662

[B68] Koziel J. Bryzek D. Sroka A. Maresz K. Glowczyk I. Bielecka E.. (2014a). Citrullination alters immunomodulatory function of LL-37 essential for prevention of endotoxin-induced sepsis. J. Immunol. 192, 5363–5372. 10.4049/jimmunol.1303062 24771854 PMC4036085

[B69] Koziel J. Mydel P. Potempa J. (2014b). The link between periodontal disease and rheumatoid arthritis: an updated review. Curr. Rheumatol. Rep. 16:408. 10.1007/s11926-014-0408-9 24458478 PMC3930831

[B70] Krauss J. L. Potempa J. Lambris J. D. Hajishengallis G. (2010). Complementary Tolls in the periodontium: how periodontal bacteria modify complement and Toll-like receptor responses to prevail in the host. Periodontol. 2000 52, 141–162. 10.1111/j.1600-0757.2009.00324.x 20017800 PMC2796596

[B71] Kumada H. Haishima Y. Umemoto T. Tanamoto K. (1995). Structural study on the free lipid A isolated from lipopolysaccharide of *Porphyromonas gingivalis*. J. Bacteriol. 177, 2098–2106. 10.1128/jb.177.8.2098-2106.1995 7721702 PMC176854

[B72] Lakschevitz F. S. Aboodi G. M. Glogauer M. (2013). Oral neutrophil transcriptome changes result in a pro-survival phenotype in periodontal diseases. PLoS ONE 8:e68983. 10.1371/journal.pone.0068983 23874838 PMC3708893

[B73] Levine A. P. Segal A. W. (2013). What is wrong with granulocytes in inflammatory bowel diseases? Dig. Dis. 31, 321–327. 10.1159/000354686 24246982 PMC4530493

[B74] Liang S. Krauss J. L. Domon H. McIntosh M. L. Hosur K. B. Qu H.. (2011). The C5a receptor impairs IL-12-dependent clearance of *Porphyromonas gingivalis* and is required for induction of periodontal bone loss. J. Immunol. 186, 869–877. 10.4049/jimmunol.1003252 21149611 PMC3075594

[B75] Ling M. R. Chapple I. L. Matthews J. B. (2015). Peripheral blood neutrophil cytokine hyper-reactivity in chronic periodontitis. Innate Immun. 21, 714–725. 10.1177/1753425915589387 26055820

[B76] Loesche W. J. Robinson J. P. Flynn M. Hudson J. L. Duque R. E. (1988). Reduced oxidative function in gingival crevicular neutrophils in periodontal disease. Infect. Immun. 56, 156–160. 3335401 10.1128/iai.56.1.156-160.1988 PMC259250

[B77] Lourbakos A. Chinni C. Thompson P. Potempa J. Travis J. Mackie E. J.. (1998). Cleavage and activation of proteinase-activated receptor-2 on human neutrophils by gingipain-R from *Porphyromonas gingivalis*. FEBS Lett. 435, 45–48. 10.1016/S0014-5793(98)01036-9 9755856

[B78] Lourbakos A. Potempa J. Travis J. D'Andrea M. R. Andrade-Gordon P. Santulli R.. (2001). Arginine-specific protease from *Porphyromonas gingivalis* activates protease-activated receptors on human oral epithelial cells and induces interleukin-6 secretion. Infect. Immun. 69, 5121–5130. 10.1128/IAI.69.8.5121-5130.2001 11447194 PMC98608

[B79] Madianos P. N. Papapanou P. N. Sandros J. (1997). *Porphyromonas gingivalis* infection of oral epithelium inhibits neutrophil transepithelial migration. Infect. Immun. 65, 3983–3990. 9316996 10.1128/iai.65.10.3983-3990.1997 PMC175572

[B80] Maekawa T. Krauss J. L. Abe T. Jotwani R. Triantafilou M. Triantafilou K.. (2014). *Porphyromonas gingivalis* manipulates complement and TLR signaling to uncouple bacterial clearance from inflammation and promote dysbiosis. Cell Host Microbe 15, 768–778. 10.1016/j.chom.2014.05.012 24922578 PMC4071223

[B81] Maresz K. J. Hellvard A. Sroka A. Adamowicz K. Bielecka E. Koziel J.. (2013). *Porphyromonas gingivalis* facilitates the development and progression of destructive arthritis through its unique bacterial peptidylarginine deiminase (PAD). PLoS Pathog. 9:e1003627. 10.1371/journal.ppat.1003627 24068934 PMC3771902

[B82] Mariano F. S. Campanelli A. P. Nociti F. H. Jr. Mattos-Graner R. O. Goncalves R. B. (2012). Antimicrobial peptides and nitric oxide production by neutrophils from periodontitis subjects. Braz. J. Med. Biol. Res. 45, 1017–1024. 10.1590/S0100-879X2012007500123 22850872 PMC3854147

[B83] Marsh P. D. (1994). Microbial ecology of dental plaque and its significance in health and disease. Adv. Dent. Res. 8, 263–271. 10.1177/08959374940080022001 7865085

[B84] Marsh P. D. (2003). Are dental diseases examples of ecological catastrophes? Microbiology 149(Pt 2), 279–294. 10.1099/mic.0.26082-0 12624191

[B85] Matthews J. B. Wright H. J. Roberts A. Cooper P. R. Chapple I. L. (2007). Hyperactivity and reactivity of peripheral blood neutrophils in chronic periodontitis. Clin. Exp. Immunol. 147, 255–264. 10.1111/j.1365-2249.2006.03276.x 17223966 PMC1810478

[B86] McCracken J. M. Allen L. A. (2014). Regulation of human neutrophil apoptosis and lifespan in health and disease. J. Cell Death 7, 15–23. 10.4137/JCD.S11038 25278783 PMC4167320

[B87] McGraw W. T. Potempa J. Farley D. Travis J. (1999). Purification, characterization, and sequence analysis of a potential virulence factor from *Porphyromonas gingivalis*, peptidylarginine deiminase. Infect. Immun. 67, 3248–3256. 10377098 10.1128/iai.67.7.3248-3256.1999 PMC116503

[B88] Mezyk-Kopec R. Bzowska M. Potempa J. Bzowska M. Jura N. Sroka A.. (2005). Inactivation of membrane tumor necrosis factor alpha by gingipains from *Porphyromonas gingivalis*. Infect. Immun. 73, 1506–1514. 10.1128/IAI.73.3.1506-1514.2005 15731048 PMC1064957

[B89] Mikolajczyk-Pawlinska J. Travis J. Potempa J. (1998). Modulation of interleukin-8 activity by gingipains from *Porphyromonas gingivalis*: implications for pathogenicity of periodontal disease. FEBS Lett. 440, 282–286. 10.1016/S0014-5793(98)01461-6 9872387

[B90] Moutsopoulos N. M. Kling H. M. Angelov N. Jin W. Palmer R. J. Nares S.. (2012). *Porphyromonas gingivalis* promotes Th17 inducing pathways in chronic periodontitis. J. Autoimmun. 39, 294–303. 10.1016/j.jaut.2012.03.003 22560973 PMC3416947

[B91] Moutsopoulos N. M. Konkel J. Sarmadi M. Eskan M. A. Wild T. Dutzan N.. (2014). Defective neutrophil recruitment in leukocyte adhesion deficiency type I disease causes local IL-17-driven inflammatory bone loss. Sci. Transl. Med. 6:229ra240. 10.1126/scitranslmed.3007696 24670684 PMC4090608

[B92] Murdock J. L. Nunez G. (2016). TLR4: the winding road to the discovery of the LPS receptor. J. Immunol. 197, 2561–2562. 10.4049/jimmunol.1601400 27638937

[B93] Murray D. A. Wilton J. M. (2003). Lipopolysaccharide from the periodontal pathogen *Porphyromonas gingivalis* prevents apoptosis of HL60-derived neutrophils *in vitro*. Infect. Immun. 71, 7232–7235. 10.1128/IAI.71.12.7232-7235.2003 14638824 PMC308905

[B94] Mydel P. Takahashi Y. Yumoto H. Sztukowska M. Kubica M. Gibson F. C. I. I. I.. (2006). Roles of the host oxidative immune response and bacterial antioxidant rubrerythrin during *Porphyromonas gingivalis* infection. PLoS Pathog. 2:e76. 10.1371/journal.ppat.0020076 16895445 PMC1522038

[B95] Nathan C. (2006). Neutrophils and immunity: challenges and opportunities. Nat. Rev. Immunol. 6, 173–182. 10.1038/nri1785 16498448

[B96] Nauseef W. M. (2007). How human neutrophils kill and degrade microbes: an integrated view. Immunol. Rev. 219, 88–102. 10.1111/j.1600-065X.2007.00550.x 17850484

[B97] Olsen I. Hajishengallis G. (2016). Major neutrophil functions subverted by *Porphyromonas gingivalis*. J. Oral Microbiol. 8:30936. 10.3402/jom.v8.30936 26993626 PMC4799392

[B98] Popadiak K. Potempa J. Riesbeck K. Blom A. M. (2007). Biphasic effect of gingipains from *Porphyromonas gingivalis* on the human complement system. J. Immunol. 178, 7242–7250. 10.4049/jimmunol.178.11.7242 17513773

[B99] Potempa J. Pike R. N. (2009). Corruption of innate immunity by bacterial proteases. J. Innate Immun. 1, 70–87. 10.1159/000181144 19756242 PMC2743019

[B100] Potempa J. Pike R. Travis J. (1997). Titration and mapping of the active site of cysteine proteinases from *Porphyromonas gingivalis* (gingipains) using peptidyl chloromethanes. Biol. Chem. 378, 223–230. 10.1515/bchm.1997.378.3-4.223 9165075

[B101] Potempa J. Sroka A. Imamura T. Travis J. (2003). Gingipains, the major cysteine proteinases and virulence factors of *Porphyromonas gingivalis*: structure, function and assembly of multidomain protein complexes. Curr. Protein Pept. Sci. 4, 397–407. 10.2174/1389203033487036 14683426

[B102] Potempa M. Potempa J. Okroj M. Popadiak K. Eick S. Nguyen K. A.. (2008). Binding of complement inhibitor C4b-binding protein contributes to serum resistance of *Porphyromonas gingivalis*. J. Immunol. 181, 5537–5544. 10.4049/jimmunol.181.8.5537 18832711 PMC2602967

[B103] Prat L. Bouaziz J. D. Wallach D. Vignon-Pennamen M. D. Bagot M. (2014). Neutrophilic dermatoses as systemic diseases. Clin. Dermatol. 32, 376–388. 10.1016/j.clindermatol.2013.11.004 24767185

[B104] Preshaw P. M. Schifferle R. E. Walters J. D. (1999). *Porphyromonas gingivalis* lipopolysaccharide delays human polymorphonuclear leukocyte apoptosis *in vitro*. J. Periodont. Res. 34, 197–202. 10.1111/j.1600-0765.1999.tb02242.x 10444743

[B105] Raetz C. R. Guan Z. Ingram B. O. Six D. A. Song F. Wang X.. (2009). Discovery of new biosynthetic pathways: the lipid A story. J. Lipid Res. 50(Suppl.), S103–S108. 10.1194/jlr.R800060-JLR200 18974037 PMC2674688

[B106] Raetz C. R. Reynolds C. M. Trent M. S. Bishop R. E. (2007). Lipid A modification systems in gram-negative bacteria. Annu. Rev. Biochem. 76, 295–329. 10.1146/annurev.biochem.76.010307.145803 17362200 PMC2569861

[B107] Rangarajan M. Aduse-Opoku J. Paramonov N. Hashim A. Bostanci N. Fraser O. P.. (2008). Identification of a second lipopolysaccharide in *Porphyromonas gingivalis* W50. J. Bacteriol. 190, 2920–2932. 10.1128/JB.01868-07 18263730 PMC2293225

[B108] Rapala-Kozik M. Bras G. Chruscicka B. Karkowska-Kuleta J. Sroka A. Herwald H.. (2011). Adsorption of components of the plasma kinin-forming system on the surface of *Porphyromonas gingivalis* involves gingipains as the major docking platforms. Infect. Immun. 79, 797–805. 10.1128/IAI.00966-10 21098107 PMC3028839

[B109] Reife R. A. Coats S. R. Al-Qutub M. Dixon D. M. Braham P. A. Billharz R. J.. (2006). *Porphyromonas gingivalis* lipopolysaccharide lipid A heterogeneity: differential activities of tetra- and penta-acylated lipid A structures on E-selectin expression and TLR4 recognition. Cell. Microbiol. 8, 857–868. 10.1111/j.1462-5822.2005.00672.x 16611234

[B110] Reynolds J. J. Meikle M. C. (1997). Mechanisms of connective tissue matrix destruction in periodontitis. Periodontol. 2000 14, 144–157. 10.1111/j.1600-0757.1997.tb00195.x 9567969

[B111] Ryder M. I. (2010). Comparison of neutrophil functions in aggressive and chronic periodontitis. Periodontol. 2000 53, 124–137. 10.1111/j.1600-0757.2009.00327.x 20403109

[B112] Saadi-Thiers K. Huck O. Simonis P. Tilly P. Fabre J. E. Tenenbaum H.. (2013). Periodontal and systemic responses in various mice models of experimental periodontitis: respective roles of inflammation duration and *Porphyromonas gingivalis* infection. J. Periodontol. 84, 396–406. 10.1902/jop.2012.110540 22655910

[B113] Sahingur S. E. Boehm T. K. Sojar H. T. Sharma A. De Nardin E. (2006). Fibrinogen-neutrophil interactions in response to fMLP and *Porphyromonas gingivalis* fimbrial peptides. Immunol. Invest. 35, 63–74. 10.1080/08820130500496811 16531330

[B114] Schwartz J. Stinson F. L. Parker R. B. (1972). The passage of tritiated bacterial endotoxin across intact gingival crevicular epithelium. J. Periodontol. 43, 270–276. 10.1902/jop.1972.43.5.270 4503430

[B115] Scott D. A. Krauss J. (2012). Neutrophils in periodontal inflammation. Front. Oral Biol. 15, 56–83. 10.1159/000329672 22142957 PMC3335266

[B116] Segal A. W. (2005). How neutrophils kill microbes. Annu. Rev. Immunol. 23, 197–223. 10.1146/annurev.immunol.23.021704.115653 15771570 PMC2092448

[B117] Settem R. P. Honma K. Sharma A. (2014). Neutrophil mobilization by surface-glycan altered Th17-skewing bacteria mitigates periodontal pathogen persistence and associated alveolar bone loss. PLoS ONE 9:e108030. 10.1371/journal.pone.0108030 25225799 PMC4167248

[B118] Sochalska M. Ottina E. Tuzlak S. Herzog S. Herold M. Villunger A. (2016). Conditional knockdown of BCL2A1 reveals rate-limiting roles in BCR-dependent B-cell survival. Cell Death Differ. 23, 628–639. 10.1038/cdd.2015.130 26450454 PMC4986635

[B119] Sochalska M. Tuzlak S. Egle A. Villunger A. (2015). Lessons from gain- and loss-of-function models of pro-survival Bcl2 family proteins: implications for targeted therapy. FEBS J. 282, 834–849. 10.1111/febs.13188 25559680 PMC4562365

[B120] Socransky S. S. Haffajee A. D. Cugini M. A. Smith C. Kent R. L. Jr. (1998). Microbial complexes in subgingival plaque. J. Clin. Periodontol. 25, 134–144. 10.1111/j.1600-051X.1998.tb02419.x 9495612

[B121] Soehnlein O. (2009). Direct and alternative antimicrobial mechanisms of neutrophil-derived granule proteins. J. Mol. Med. 87, 1157–1164. 10.1007/s00109-009-0508-6 19641860

[B122] Summers C. Rankin S. M. Condliffe A. M. Singh N. Peters A. M. Chilvers E. R. (2010). Neutrophil kinetics in health and disease. Trends Immunol. 31, 318–324. 10.1016/j.it.2010.05.006 20620114 PMC2930213

[B123] Takeuchi H. Hirano T. Whitmore S. E. Morisaki I. Amano A. Lamont R. J. (2013). The serine phosphatase SerB of *Porphyromonas gingivalis* suppresses IL-8 production by dephosphorylation of NF-kappaB RelA/p65. PLoS Pathog. 9:e1003326. 10.1371/journal.ppat.1003326 23637609 PMC3630210

[B124] Tuzlak S. Kaufmann T. Villunger A. (2016). Interrogating the relevance of mitochondrial apoptosis for vertebrate development and postnatal tissue homeostasis. Genes Dev. 30, 2133–2151. 10.1101/gad.289298.116 27798841 PMC5088563

[B125] Van Dyke T. E. Sheilesh D. (2005). Risk factors for periodontitis. J. Int. Acad. Periodontol. 7, 3–7. 15736889 PMC1351013

[B126] Vier J. Groth M. Sochalska M. Kirschnek S. (2016). The anti-apoptotic Bcl-2 family protein A1/Bfl-1 regulates neutrophil survival and homeostasis and is controlled via PI3K and JAK/STAT signaling. Cell Death Dis. 7:e2103. 10.1038/cddis.2016.23 26890142 PMC5399193

[B127] Vincents B. Guentsch A. Kostolowska D. von Pawel-Rammingen U. Eick S. Potempa J.. (2011). Cleavage of IgG1 and IgG3 by gingipain K from *Porphyromonas gingivalis* may compromise host defense in progressive periodontitis. FASEB J. 25, 3741–3750. 10.1096/fj.11-187799 21768393 PMC3177567

[B128] Wang M. Shakhatreh M. A. James D. Liang S. Nishiyama S. Yoshimura F.. (2007). Fimbrial proteins of porphyromonas gingivalis mediate *in vivo* virulence and exploit TLR2 and complement receptor 3 to persist in macrophages. J. Immunol. 179, 2349–2358. 10.4049/jimmunol.179.4.2349 17675496

[B129] White P. C. Chicca I. J. Cooper P. R. Milward M. R. Chapple I. L. (2016). Neutrophil extracellular traps in periodontitis: a web of intrigue. J. Dent. Res. 95, 26–34. 10.1177/0022034515609097 26442948

[B130] White P. Sakellari D. Roberts H. Risafi I. Ling M. Cooper P.. (2016). Peripheral blood neutrophil extracellular trap production and degradation in chronic periodontitis. J. Clin. Periodontol. 43, 1041–1049. 10.1111/jcpe.12628 27678376

[B131] Wiesmeier M. Gautam S. Kirschnek S. Hacker G. (2016). Characterisation of neutropenia-associated neutrophil elastase mutations in a murine differentiation model *in vitro* and *in vivo*. PLoS ONE 11:e0168055. 10.1371/journal.pone.0168055 27942017 PMC5152902

[B132] Wingrove J. A. DiScipio R. G. Chen Z. Potempa J. Travis J. Hugli T. E. (1992). Activation of complement components C3 and C5 by a cysteine proteinase (gingipain-1) from Porphyromonas (Bacteroides) gingivalis. J. Biol. Chem. 267, 18902–18907. 1527018

[B133] Yasunari K. Watanabe T. Nakamura M. (2006). Reactive oxygen species formation by polymorphonuclear cells and mononuclear cells as a risk factor of cardiovascular diseases. Curr. Pharm. Biotechnol. 7, 73–80. 10.2174/138920106776597612 16724940

[B134] Yu J. J. Ruddy M. J. Wong G. C. Sfintescu C. Baker P. J. Smith J. B.. (2007). An essential role for IL-17 in preventing pathogen-initiated bone destruction: recruitment of neutrophils to inflamed bone requires IL-17 receptor-dependent signals. Blood 109, 3794–3802. 10.1182/blood-2005-09-010116 17202320 PMC1874584

[B135] Zaric S. Shelburne C. Darveau R. Quinn D. J. Weldon S. Taggart C. C.. (2010). Impaired immune tolerance to *Porphyromonas gingivalis* lipopolysaccharide promotes neutrophil migration and decreased apoptosis. Infect. Immun. 78, 4151–4156. 10.1128/IAI.00600-10 20679442 PMC2950342

